# Characteristics of the mitochondrial genome of *Rana omeimontis* and related species in Ranidae: Gene rearrangements and phylogenetic relationships

**DOI:** 10.1002/ece3.6824

**Published:** 2020-10-31

**Authors:** Lichun Jiang, Min Zhang, Lu Deng, Zhongwen Xu, Hongyan Shi, Xiaodong Jia, Zhenli Lai, Qiping Ruan, Wei Chen

**Affiliations:** ^1^ Key Laboratory for Molecular Biology and Biopharmaceutics School of Life Science and Technology Mianyang Normal University Mianyang China; ^2^ Ecological Security and Protection Key Laboratory of Sichuan Province Mianyang Normal University Mianyang China

**Keywords:** control region, gene rearrangements, mitochondrial genome, phylogeny, protein‐coding genes, *Rana omeimontis*, Ranidae

## Abstract

The Omei wood frog (*Rana omeimontis*), endemic to central China, belongs to the family Ranidae. In this study, we achieved detail knowledge about the mitogenome of the species. The length of the genome is 20,120 bp, including 13 protein‐coding genes (PCGs), 22 tRNA genes, two rRNA genes, and a noncoding control region. Similar to other amphibians, we found that only nine genes (ND6 and eight tRNA genes) are encoded on the light strand (L) and other genes on the heavy strand (H). Totally, The base composition of the mitochondrial genome included 27.29% A, 28.85% T, 28.87% C, and 15.00% G, respectively. The control regions among the *Rana* species were found to exhibit rich genetic variability and A + T content. *R. omeimontis* was clustered together with *R. chaochiaoensis* in phylogenetic tree. Compared to *R. amurensis* and *R. kunyuensi*, it was more closely related to *R. chaochiaoensis*, and a new way of gene rearrangement (ND6‐trnE‐Cytb‐D‐loop‐trnL2 (CUN)‐ND5‐D‐loop) was also found in the mitogenome of *R. amurensis* and *R. kunyuensi*. Our results about the mitochondrial genome of *R. omeimontis* will contribute to the future studies on phylogenetic relationship and the taxonomic status of *Rana* and related Ranidae species.

## Graphical abstract

The Omei wood frog (*Rana omeimontis*), endemic to central China, belongs to the family Ranidae. *R. omeimontis* was clustered together with R. chaochiaoensis in phylogenetic tree. Compared to *R. amurensis* and *R. kunyuensi*, it was more closely related to *R. chaochiaoensis*, and a new way of gene rearrangement (ND6‐ trnE‐ Cytb‐ D‐ loop‐ trnL2 (CUN) ‐ ND5‐ D‐ loop) was also found in the mitogenome of *R. amurensis* and *R. kunyuensi*. Our results about the mitochondrial genome of *R. omeimontis* will contribute to the future studies on phylogenetic relationship and the taxonomic status of Rana and related Ranidae species.

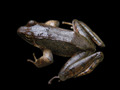

## 1 INTRODUCTION

The *Rana omeimontis* is a member of the Ranidae family species and is endemic to the central part of China (Sichuan, southern Gansu, western Hunan, southwestern Hubei and Guizhou). Its natural habitats are hill forests and grass clumps in forests with an elevation of 520–2,100 m (Michae & Zhao, 2004). Females have larger body size than males (Fei, 1999). The *R. omeimontis* was listed as threatened species in the IUCN after 2004 (Michae & Zhao, 2004). Major threats to the species include habitat destruction and degradation, dam construction, and water pollution. Luckily, main distribution range of the frogs is located in some nature reserves (Michae & Zhao, 2004).

Vertebrate mtDNAs are known as closed circular structure. The mitogenomes spread a range of 15 to 21 kb (Ni et al., 2016; Oliver et al., 2015; Sano et al., 2005). The mitochondria in eukaryotic cells are very significant functional organelles (Koehler & Bauer, 2004). MtDNA, as an ideal molecular marker, has been widely used for molecular evolution and phylogenetic status because of obvious benefits including its simple genomic arrangement, high richness, small size, rapid evolutionary rate, low levels of sequence recombination, high mutation rate, and clear orthology. (Boore, 2006; Mu et al., 2012, 2015; Wang et al., 2020; Yang et al., 2018; Zhang et al., 2015).

From fish to mammals, mitochondrial gene arrangement has been proved to be conserved (Boore, 1999; Irisarri et al., 2012; Kurabayashi et al., 2010; San Mauro et al., 2004), while for neobatrachian species, gene rearrangement is more common (Duellman, 2003; Xia et al., 2014). However, for some neobatrachian taxon, the gene arrangement is remarkably different with four tRNA gene clusters (trnL2‐trnT‐trnP‐trnF clusters, LTPF clusters) usually rearranged in this groups (Irisarri et al., 2012; Zhang et al., 2005). The mitochondrial gene orders show especially high divergence in some species including Mantellidae (Kurabayashi et al., 2006), Rhacophoridae (Ren et al., 2009; Sano et al., 2004, 2005) and Dicroglossidae (Jing et al., 2020; Li et al., 2014; Yuan et al., 2016; Zhang et al., 2018). Gene rearrangements are common in vertebrate mitochondrial genomes, and these derived gene rearrangements can be used as phylogenetic makers (Irisarri et al., 2012; Kurabayashi et al., 2008; San Mauro et al., 2006). So far, the ranid mitogenomes, unlike these natatanurans species, exhibit the typical neobatrachian‐type gene arrangements and a stable mtNDA gene arrangement pattern has been observed among the Ranidae species (Atsushi & Masayuki, 2013; Kurabayashi et al., 2006; Ren et al., 2009; San Mauro et al., 2006). Little is unknown about the gene rearrangement types and rearrangement mechanism of the *Rana* and ranid species, and these mechanism need further analysis and discussion.

The taxonomy of ranid species is complex, and it still remains controversial (Che et al., 2007; Chen et al., 2005; Dubois, 2005; Frost et al., 2006; Jiang & Zhou, 2005; Matsui et al., 2006). Monophyly of the high‐level taxa remains to be tested phylogenetically (Dubois, 2005). Recently, combined the anatomical and genetical data, Frost et al. (2006) proposed new classification system for amphibians, and Raninae including 18 generic taxa was elevated to family status (Dubois, 2005; Frost, 2006). Yet monophylies of three genera (*Rana*, *Amolops*, and *Pseudoamolops*) was not supported (Dubois, 2005; Marmayou et al., 2000). These revisions were based on genetic diversity of these frogs, especially for the Chinese species (Che et al., 2007). Although some molecular phylogenetic analysis on these taxa has been recorded (Bossuyt et al., 2006; Hillis & Wilcox, 2005; Matsui et al., 2006), the detail phylogenetic systematics for these taxa remains unclear. However, to gain a robust phylogeny for the family Ranidae, intensive taxa samplings are in need.

To date, there are some researches of other species of *Rana*, but the characteristics of mitogenome and phylogenetic knowledge of *R. omeimontis* has been reported less. In this study, we detaily explored the *R. omeimontis* mitogenome characteristics and its evolution status. We focused on more extensive classification samplings within *Rana* and Ranidae, and sequences from GenBank were also included in our analysis using mitogenome data. We explored mitogenome characteristics, phylogenetic relationships, and gene rearrangement mechanisms in Ranidae.

## 2 MATERIALS AND METHODS

### 2.1 Sampling and DNA extraction

**FIGURE 1:**
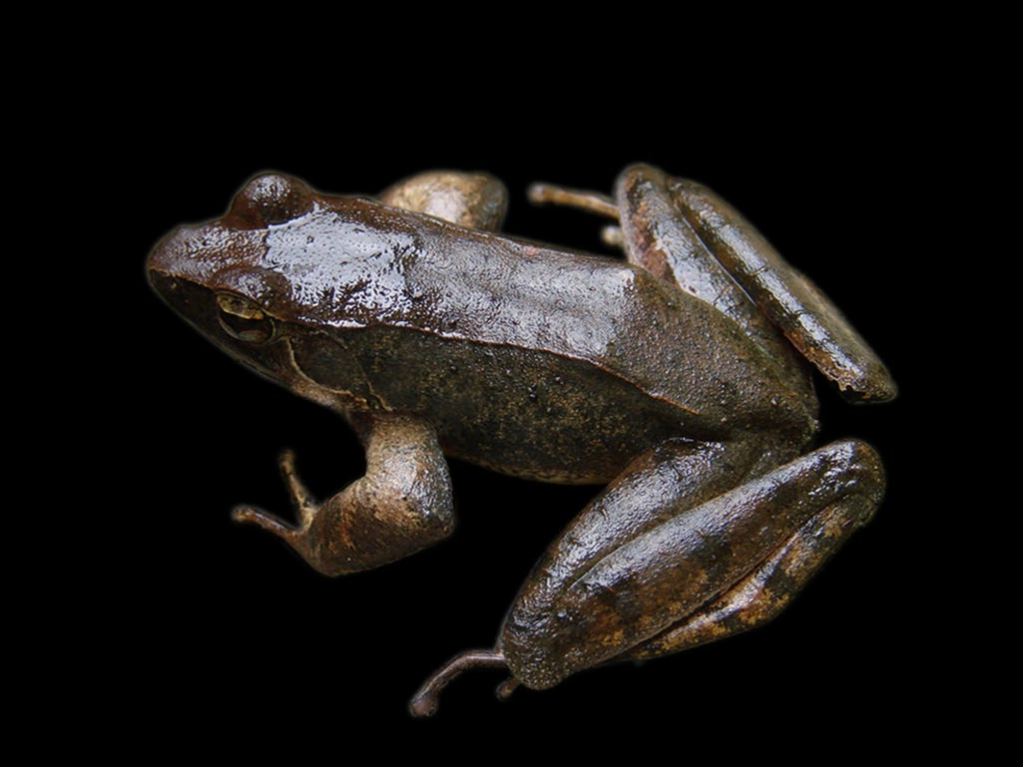
The Omei wood frog, *Rana omeimontis* was collected from Mount Emei, Sichuan Province

**FIGURE 2:**
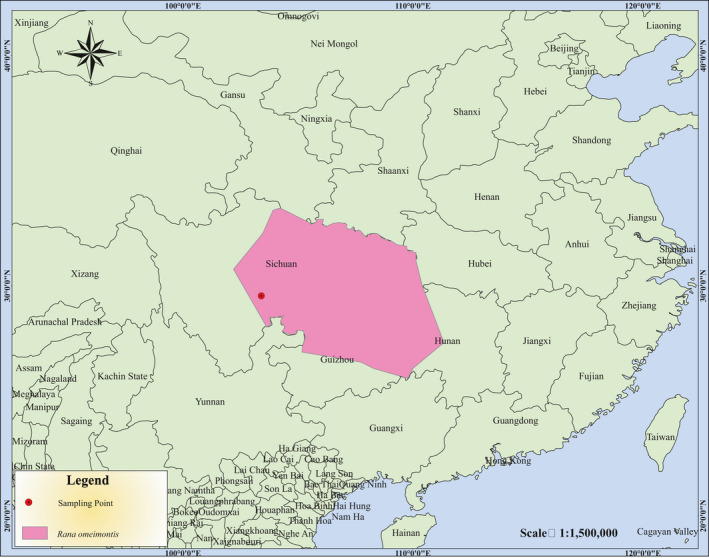
Species distribution map of the Omei wood frog, *Rana omeimontis*

The Omei wood frog, *R. omeimontis* (Figure 1), was collected from the Shengshui temple (103°24′32.59″E, 29°33′49.66″N), Mount Emei, Sichuan Province, China in June 2018. The distribution of this species is shown in Figure 2. All the experimental protocols and methods were carried out under the rules and regulations of the Academic Research Steering Committee of Mianyang Normal University and the requirements of the ethics committee of Mianyang Normal University. The webbed feet of the frog were clipped and preserved in ethanol (95%) and then stored at −70°C. According to the manufacturer's instruction, whole genomic DNA from two adults was extracted using the protocol of Tissue DNA Kit (Omega, USA) and diluted to 50 ng%μl for polymerase chain reaction (PCR) (Jiang et al., 2013).

### 2.2 Mitochondrial DNA amplification and sequencing

The total mitogenome was amplified 15 overlapping segments with LA and rTaq DNA Polymerase (TaKaRa Co, China) and 30 ng of genomic DNA as template. To amplify the complete mitogenome sequence, 15 pairs of primers were adopted, of which 8 pairs were derived from the literature (Kurabayashi & Sumida, 2009), and the other 7 pairs were designed based on their own the relatively conserved regions of its congeneric *R. chaochiaoensis* (NC_035803) and *R. zhenhaiensis* (MF370348). PCR amplification was carried out according to the methods adopted by Jiang et al. (2017), Jiang et al. (2018). Specifically, a total volume of 50 μl with 0.4 μl of LA Taq or rTaq, 3.0 μl of DNA, 4.0 μl dNTPs, 5.0 μl 25 mM MgCl_2,_ and 2.0 μl 2.5 μM primers were used for PCR amplification. The reaction protocol was as follows: initial denaturation step at 94°C for 3.5 min; subsequent 32 cycles of denaturation at 94°C (40 s), then annealing of the primers at 50–62°C (30 s), next extension at 62°C (90–300 s), and ending with a final extension at 72°C for 9 min. The PCR segments were detected by gel electrophoresis and purified with the E.Z.N.A Gel Extraction Kit (Omega, USA). Finally, direct sequencing was performed with an ABI 3730 sequencer. To improve its accuracy, all PCR fragments were sequenced twice.

### 2.3 Sequence assembling and analysis

PCR segments were assembled with the Staden Package v1.7.0 (Staden et al., 2000), and the amino acid sequences were aligned in Clustal X 1.83 (Thompson et al., 1997). The mitogenome sequence of *R. omeimontis* has been submitted to NCBI GenBank library (accession number MK483118). 13 PCGs were translated into its corresponding amino acid sequences with MEGA 6.06 software (Tamura et al., 2013). We gained the base composition and relative synonymous codon usage (RSCU) in MEGA 6.06 (Tamura et al., 2013). Two rRNAs were confirmed according to BLAST search in NCBI. And we achieved their secondary structures according to XRNA 1.2.0.b program (Cannone et al., 2002). The secondary structure of 22 tRNAs was recognized with tRNAscan‐SE 1.21 and ARWEN software (Laslett & Canback, 2008; Lowe & Eddy, 1997) with the default setting. Nucleotide compositional skew analysis was executed on the basis of two formulas: AT‐skew = (A − T)%(A + T) and GC‐skew = (G − C)%(G + C), respectively (Perna & Kocher, 1995).

In addition, we hand‐counted the gene overlap and intergenic‐space sequences. We also identified origin of light‐strand replication (O_L_) and control region through comparison with the homologous sequences of other closely related wood frogs, and achieved the secondary structure of the putative O_L_ using Mfold v.3.2 (http://mfold.bioinfo.rpi.edu/) (Zuker, 2003) and visualized using RNAViz (De Rijk & Wachter, 1997).

### 2.4 Phylogenetic analysis

Phylogenetic trees were reconstructed for ranid frogs using Bayesian inference (BI) and maximum likelihood (ML) analyses with the two combined gene sets. Two datasets were generated for phylogenetic reconstruction: (a) P13: 13 PCGs (11,235 bp), all termination codons and ambiguous sites of 13 PCGs were manually deleted; (b) P13RT: 13 PCGs + 2 rRNAs + 21 tRNAs (15,106 bp). Phylogenetic analyses were performed according to 48 complete mitogenomes (Table 1). To clarify the evolutionary relationship of *R. omeimontis*, all available and complete mitogenomes of Ranidae were used, with *Microhylaornata* (NC_009422) and *Paa spinosa* (FJ432700) as outgroups. The 13 PCGs and complete mitogenome sequences were aligned in Clustal X 1.83 (Thompson et al., 1997). Using the ML and BI methods, phylogenetic trees were constructed. The best fitting models were chosen in jModeltest v.0.1.1 (Posada, 2008; Posada& Buckley, 2004). BI was performed in MrBayes 3.2.2 (Ronquist et al., 2012). According to the AIC, the best fitting models (the GTR + I+G) of nucleotide datasets were chosen (Lanave et al., 1984). BI analyses as the following conditions: under Markov Chain Monte Carlo (MCMC) assessment, four chains (one hot chain and three cold chains) were set to run synchronously for 8,000,000 generations, the MCMC analyses were executed to estimate the consistency of posterior distributions and the trees were sampled every 1,000 generations with a burn‐in step. The confidence values for the BI tree were estimated as the Bayesian posterior probabilities (BPP) in percentages, and BPP over 0.9 were regarded as powerfully supported. ML analysis was carried out in PhyML package (v.3.0; Guindon & Gascuel, 2003). In ML analysis, the confidence level was counted using four substitution rate categories and bootstrap replicates of 1,000 (Felsenstein, 1985).

**TABLE 1:** Mitogenomes of the Ranidae used in this study

Family	Genus	Species	Size (bp)	Accession no.	Reference
Ranidae	*Rana*	*Rana omeimontis*	20,210	**MK483118**	**This study**
		*Rana omeimontis*	19,934	KU246050	Yang et al. (2018)
		*Rana draytonii*	17,805	KP013110	Genbank
		*Rana dybowskii*	18,864	NC_023528	Li et al. (2014)
		*Rana huanrensis*	19,253	NC_028521	Dong et al. (2016)
		*Rana kunyuensis*	22,255	NC_024548	Li et al. (2014)
		*Rana cf. chensinensis*	18,808	NC_023529	Li et al. (2014)
		*Rana catesbeiana*	18,241	NC_022696	Lin et al. (2014)
		*Rana sylvatica*	17,343	NC_027236	Ni et al. (2016)
		*Rana okaloosae*	17,504	NC_028283	Genbank
		*Rana amurensis*	20,571	MF370348	Liu et al. (2017)
		*Rana kukunoris*	18,863	NC_035804	Liu et al. (2017)
		*Rana pyrenaica*	17,211	KU720300	Peso‐Fernandez et al. (2016)
		*Rana chaochiaoensis*	18,591	NC_035803	Liu et al. (2017)
	*Amolops*	*Amolops wuyiensis*	17,308	KM282625	Zhang et al. (2018)
		*Amolops mantzorum*	17,744	NC_024180	Su et al. (2007)
		*Amolops ricketti*	17,772	KF956111	Li et al. (2014)
		*Amolops loloensis*	18,926	NC_029250	Xue et al.(2016)
	*Odorrana*	*Odorrana ishikawae*	21,020	NC_015305	Kurabayashi et al.(2010)
		*Odorrana tormotus*	17,962	DQ835616	Su et al. (2007)
		*Odorrana wuchuanensis*	18,256	NC_034983	Huang et al. (2017)
		*Odorrana margaretae*	17,903	NC_024603	Chen et al. (2015)
		*Odorrana hainanensis*	17,986	NC_034984	Huang et al. (2017)
		*Odorrana schmackeri*	18,610	KP732086	Bu et al. (2016)
		*Odorrana livida*	16,057	NC043768	Zhang et al. (2018)
	*Pelophylax*	*Pelophylax chosenica*	18,357	NC_016059	Ryu and Hwang (2007)
		*Pelophylax plancyi*	17,822	NC_009264	Genbank
		*Pelophylax nigromaculata*	17,804	NC_002805	Sumida et al.(2001)
		*Pelophylax nigromaculatus*	17,567	KT878718	Jiang et al. (2017)
		*Pelophylax bedriagae*	17,968	KP260932	Genbank
		*Pelophylax cf. terentievi*	17,990	KP260931	Genbank
		*Pelophylax shqipericus*	17,366	NC_026896	Hofman et al. (2016)
		*Pelophylax kurtmuelleri*	18,020	NC_026895	Hofman et al. (2016)
		*Pelophylax epeiroticus*	18,030	NC_026894	Hofman et al. (2016)
		*Pelophylax cypriensis*	18,023	NC_026893	Hofman et al. (2016)
		*Pelophylax* sp. GM4−14	17,939	KP260933	Genbank
		*Pelophylax cretensis*	17,829	KM677928	Hofman et al. (2016)
	*Amnirana*	*Amnirana albolabris*	15,171	JX564871	Zhang et al. (2013)
	*Hylarana*	*Hylarana guentheri*	19,053	NC_024748	Wu et al. (2016)
	*Babina*	*Babina adenopleura*	18,982	JX033120	Yu et al. (2012 **)**; Yu et al. (2012)
		*Babina holsti*	19,113	NC_022870	Kakehashi et al.(2013)
		*Babina okinavana*	19,959	NC_022872	Kakehashi et al.(2013)
		*Babina subaspera*	18,525	NC_022871	Kakehashi et al.(2013)
	*Glandirana*	*Glandirana emeljanovi*	17,733	NC_030211	Liu et al. (2017)
		*Glandirana emeljanovi*	19,294	KF771343	Xia et al.(2014)
		*Glandirana rugosa*	17,426	KF771341	Xia et al.(2014)
		*Glandirana tientaiensis*	17,347	KF771342	Xia et al.(2014)
		*Glandirana tientaiensis*	17,681	NC_025226	Yan et al. (2016)
Microhylidae	*Microhyla*	*Microhyla ornata*	16,730	NC_009422	Genbank
Dicroglossidae	*Quasipaa*	*Paa spinosa*	18,012	FJ432700	Zhou et al. (2009)

This bold represents the sequence obtained in this study.

## 3 RESULTS

### 3.1 Genome content and organization

**FIGURE 3:**
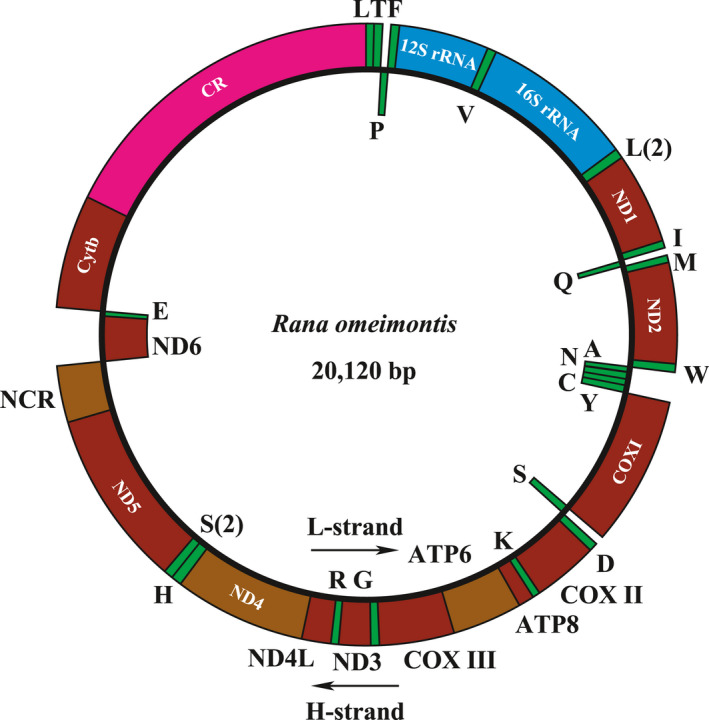
Complete mitochondrial genome organization and gene arrangement of *Rana omeimontis*. Genes coded on the H‐strand are directed to the outer ring, while the genes coded on the L‐strand are indicated in the interior of the ring. Genes are abbreviated as follows: ATP6 and ATP8 (subunits 6 and 8 of ATPase), COI‐COIII (cytochrome c oxidase subunits 1–3), Cytb (cytochrome b), ND1‐ND6 and ND4L (NADH dehydrogenase subunits 1–6 and 4L), 12S rRNA and 16S rRNA (ribosomal RNA of 12S and 16S), CR (control region, D‐loop), and NCR (Noncoding region). One‐letter amino acid abbreviations were used to label the corresponding tRNA genes. The arrow represents the direction of transcription

The whole mitogenome sequence of *R. omeimontis* is a closed circular structure 20,120 bp in length, containing 13 PCGs, 2 rRNA genes (12S rRNA and 16S rRNA), 22 tRNA genes, and a D‐loop (CR). The general characteristics of mitochondrial genomes in Ranidae are listed in Table 1. These complete mitogenomes range from 17,211 to 22,255 bps. Length differences mainly result from the variation in lengths and%or numbers or repeated sequence times of the control region. The mitogenome demonstrates the typical gene content observed in vertebrate mitogenomes, gene locations are shown in Figure 3 and Table 2. There are 9 genes encoded on L‐strand (ND6 and eight tRNAs) and 29 genes on H‐strand (14 tRNAs, two rRNAs, 12 PCGs and a D‐loop). The organization of the gene sequence for *R. omeimontis* is in consistence with other ranids. The base composition of nucleotide sequences of the entire mitochondrial genome is A:27.29%; T(U): 28.85%; C: 28.87%; G: 15.00%. The content of A + T is 56.14%, suggesting a A‐ and T‐biased, which similarly to other vertebrate mtDNAs (Igawa et al., 2008; Li, et al., 2016; Li, et al., 2016).

**TABLE 2:** Characteristics of the mitochondrial genome of *Rana omeimontis*

Gene	Position	Sizes	Codon	Intergenic	Strand^c^	A + T%		
	from	to	Nudeotide (bp)	start	stop^a^	Nudeotide^b^		
tRNA‐Leu2	1	72	72			2	H	51.39
tRNA‐Thr	75	144	70			0	H	51.43
tRNA‐Pro	145	213	69			1	L	59.42
tRNA‐Phe	215	284	70			0	H	65.71
12S ribosomal RNA	285	1,216	932			−1	H	53.54
tRNA‐Val	1,216	1,284	69			0	H	59.42
16S ribosomal RNA	1,285	2,862	1578			1	H	57.73
tRNA‐Leu	2,864	2,936	73			6	H	60.27
ND1	2,940	3,897	958	AAA	T‐‐	0	H	55.22
tRNA‐Ile	3,898	3,968	71			0	H	45.07
tRNA‐Gln	3,969	4,039	71			−1	L	66.20
tRNA‐Met	4,039	4,107	69			0	H	62.32
ND2	4,108	5,142	1,035	ATG	TAG	−2	H	54.98
tRNA‐Trp	5,141	5,210	70			0	H	57.14
tRNA‐Ala	5,211	5,280	70			0	L	60.00
tRNA‐Asn	5,281	5,353	73			0	L	57.53
rep‐origin	5,354	5,384	31			−3	L	51.61
tRNA‐Cys	5,382	5,446	65			0	L	44.62
tRNA‐Tyr	5,447	5,513	67			4	L	46.27
COI	5,518	7,068	1551	ATA	AGG	−9	H	53.19
tRNA‐Ser2	7,060	7,130	71			1	L	46.48
tRNA‐Asp	7,132	7,200	69			0	H	60.87
COII	7,201	7,888	688	ATG	T‐‐	0	H	54.22
tRNA‐Lys	7,889	7,957	69			1	H	60.87
ATP8	7,959	8,120	162	ATG	TAA	−23	H	59.88
ATP6	8,114	8,795	682	GTG	T‐‐	0	H	56.01
COIII	8,796	9,579	784	ATG	T‐‐	0	H	51.91
tRNA‐Gly	9,580	9,647	68			0	H	64.71
ND3	9,648	9,987	340	ATG	T‐‐	0	H	54.12
tRNA‐Arg	9,988	10,057	69			0	H	58.57
ND4L	10,058	10,342	285	GTG	TAA	−7	H	52.63
ND4	10,336	11,695	1,360	ATG	T‐‐	0	H	54.52
tRNA‐His	11,696	11,764	69			0	H	65.22
tRNA‐Ser	11,765	11,831	67			30	H	49.25
ND5	11,862	13,649	1788	ATG	AGA	0	H	54.63
misc_feature Noncoding region	13,650	14,200	551			0	H	
ND6	14,201	14,695	495	ATG	AGA	0	L	51.92
tRNA‐Glu	14,696	14,764	69			3	L	56.52
Cytb	14,768	15,910	1,143	ATG	TAA	0	H	51.09
D‐loop	15,911	20,120	4,210			0	H	62.92

^a^ T—represent incomplete stop codons.

^b^ Numbers correspond to the nucleotides separating adjacent genes, negative numbers indicate overlapping nucleotides.

^c^ H and L indicate genes transcribed on the heavy and light strands, respectively.

As for intergenic spacers and overlap region, there are 49 nucleotides dispersing in 9 intergenic spacers from 1 to 30 bp and 46 bases of overlapping genes at 7 boundaries with a range of 1 to 23 bp in the whole mtDNA of *R. omeimontis*. The longest intergenic spacer region is 30 nucleotides, which is located between ND5 and trnS2 and overlapping segments (23bp) existed between ATP8 and ATP6, while the shortest overlap (1bp) in 12S rRNA‐trnV and trnQ‐trnM, respectively (Table 2).

### 3.2 Protein‐coding genes and codon usages

The size of 13 protein‐coding genes (PCGs) in *R. omeimontis* mitogenome is 11,265 bp, which were accounting for 55.99% in the total mitogenome sequence (Table 2). The base composition for the total 13 PCGs in the *R. omeimontis* mitogenome (A:24.35%; T:29.63%; C:30.08%; G:15.94%) are shown in Table S1. A + T content in the total 13 PCGs is 53.98%. Three codon positions and compositions of the total 13 PCGs are shown in Table S1, the A + T content of the first, second, and third positions are 59.91%, 59.23%, and 52.81%, respectively. The skewness of the base composition in nucleotide sequences is used to investigate the relative numbers of A to T (AT‐skew) and G to C (GC‐skew). The results of the nucleotide skew statistics exhibit that the AT skewness of the first (0.07) and the third positions (0.08) are slightly positive, and the second position is obviously negative (−0.57), while the CG skewness of three positions is negative (Table S1). The pattern of nucleotide skewness in *R. omeimontis* mitochondrial genomes is consistent with that of most other *Rana* species (Huang et al., 2019; Wang et al., 2020). The A + T content for each of PCGs in the *R. omeimontis* mitogenomes is presented in Table S2, ATP8 has the highest A + T content (59.88%), and moreover, the lowest is 51.10% in Cytb.

The start and stop codons of the 13 PCGs are shown in Table 2. There are 9 genes (ATP8, COII‐III, ND2‐6, and Cytb) take advantage of the ATG as start codon. Nevertheless, the other genes are not the same again. The ND4L and ATP6 start with GTG; the ND1 and COI genes start with AAA and ATA, respectively. As for stop codons, six PCGs (ND1, ND3, ND4, COII, COIII, and ATP6) terminate with a single T residue (Table 2) which is completed (TAA) through polyadenylation during transcription processing (Boore, 2001). Three PCGs (ATP8, ND4L, and Cytb) are terminated with TAA. The ND2 and COI stop with TAG and AGG, respectively, while the ND5 and ND6 utilized AGA as stop codon (Table 2).

Codon usage of 13 PCGs is presented in Table 3. There are 3,755 codons in total which constitute the 13 PCGs. Among the 64 available codons, the CUC (4.47%), AUC (4.15%), GCC (4.15%), UUC (3.60%), and CUA (3.60%) are the five most frequent codons. Whereas the CGG, CGU, AAG, CCG, and UGU codons are seldom represented, all of these accounted for 1.09%. Leu, Ile, Phe, and Ala are the richest amino acids in all the PCGs of *R. omeimontis* mitogenome (Table 3).

**TABLE 3:** Codon usage in *Rana omeimontis* mitochondrial protein‐coding genes

Codon	Count	RSCU	Codon	Count	RSCU	Codon	Count	RSCU	Codon	Count	RSCU
UUU(F)	127	0.97	UCU(S)	65	1.47	UAU(Y)	48	0.83	UGU(C)	**10**	0.67
UUC(F)	**135**	1.03	UCC(S)	72	1.62	UAC(Y)	67	1.17	UGC(C)	20	1.33
UUA(L)	110	1.05	UCA(S)	66	1.49	UAA(*)	3	1.71	UGA(W)	77	1.43
UUG(L)	29	0.28	UCG(S)	10	0.23	UAG(*)	1	0.57	UGG(W)	31	0.57
CUU(L)	124	1.18	CCU(P)	47	0.91	CAU(H)	28	0.53	CGU(R)	**8**	0.43
CUC(L)	**168**	1.6	CCC(P)	101	1.96	CAC(H)	77	1.47	CGC(R)	25	1.35
CUA(L)	**135**	1.28	CCA(P)	49	0.95	CAA(Q)	65	1.63	CGA(R)	36	1.95
CUG(L)	65	0.62	CCG(P)	**9**	0.17	CAG(Q)	15	0.38	CGG(R)	**5**	0.27
AUU(I)	129	0.91	ACU(T)	65	0.89	AAU(*N*)	60	0.92	AGU(S)	17	0.38
AUC(I)	**156**	1.09	ACC(T)	111	1.52	AAC(*N*)	70	1.08	AGC(S)	36	0.81
AUA(M)	101	1.29	ACA(T)	101	1.38	AAA(K)	75	1.79	AGA(*)	2	1.14
AUG(M)	55	0.71	ACG(T)	15	0.21	AAG(K)	**9**	0.21	AGG(*)	1	0.57
GUU(V)	50	0.93	GCU(A)	87	1.06	GAU(D)	26	0.72	GGU(G)	28	0.5
GUC(V)	67	1.25	GCC(A)	**156**	1.91	GAC(D)	46	1.28	GGC(G)	86	1.53
GUA(V)	63	1.18	GCA(A)	64	0.78	GAA(E)	64	1.49	GGA(G)	53	0.94
GUG(V)	34	0.64	GCG(A)	20	0.24	GAG(E)	22	0.51	GGG(G)	58	1.03

### 3.3 Transfer and ribosomal RNA genes

**FIGURE 4:**
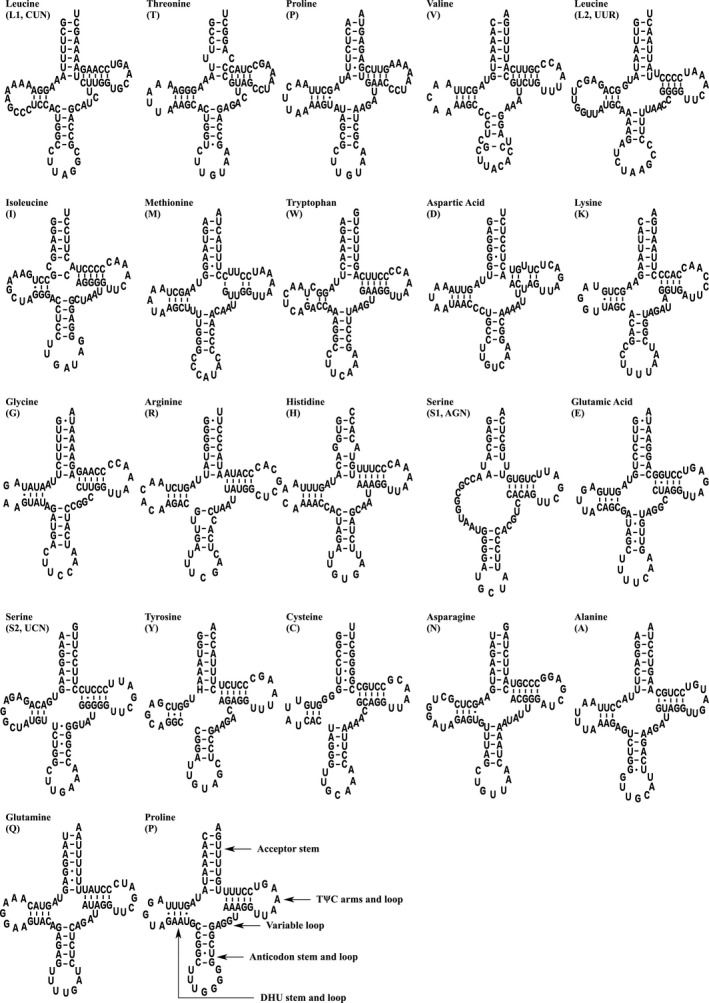
Putative tRNA secondary structures predicted from the 22 tRNA gene sequences found in the *Rana omeimontis* mitochondrial genome

22 tRNA genes scattered in the mitogenome of *R. omeimontis*, with 65 bp for trnC to 73 bp for trnN and trnL2. 8 of 22 genes are on L‐strand, while the remains are on H‐strand. All the tRNAs in *R. omeimontis*, with exception of the trnS2 (AGY), where the DHU arm was substituted by eleven unpaired nucleotides, fold into classic canonical cloverleaf secondary structure. Besides, there were 40 non‐Watson–Crick base pairs in 22 tRNAs (Figure 4). There are thirty G‐U pairs and the atypical ten pairs (three U‐C, four U‐U, two A‐C, one A‐G) unmatched were observed in trnT (receptor arm, one U‐C), trnV (anticodon arm, one U‐C), trnR (anticodon arm, one U‐C), trnD (TΨC arm, one U‐U), trnM (TΨC arm, two U‐U), trnS (receptor arm, one U‐U), trnW (dihydrouracil arm, one A‐C), trnI (receptor arm, one A‐C), and trnH (receptor arm, one A‐G), respectively.

The 12S rRNA and 16S rRNA were located between trnF and trnL (UUR) genes and separated by the trnV gene. The size of 12S rRNA is 932 bp while the size of 16S rRNA is 1,578 bp. The composition of nucleotides of 12S rRNA is 23.93% T(U), 26.39% C, 29.61% A, and 20.06% G. And the composition of nucleotides of 16S rRNA is 23.70% T(U), 24.71% C, 34.03% A, and 17.55% G.

### 3.4 Noncoding regions

O_L_ region with 31 nucleotides was located in within the 5 tRNA gene clusters (Figure S1). This region could form a stem‐loop structure with nine or ten paired nucleotides in stem and 10 or 11 nucleotides in loop (Figure S1). A conserved sequence (5'‐GCCGG‐3') in the trnC gene can be found to participated in DNA synthesis from RNA (Figure S1, Hixson & Brown, 1986). The comparatively conserved stem segment and highly variable loop sequence of O_L_ were also observed (Figure S1).

**FIGURE 5:**
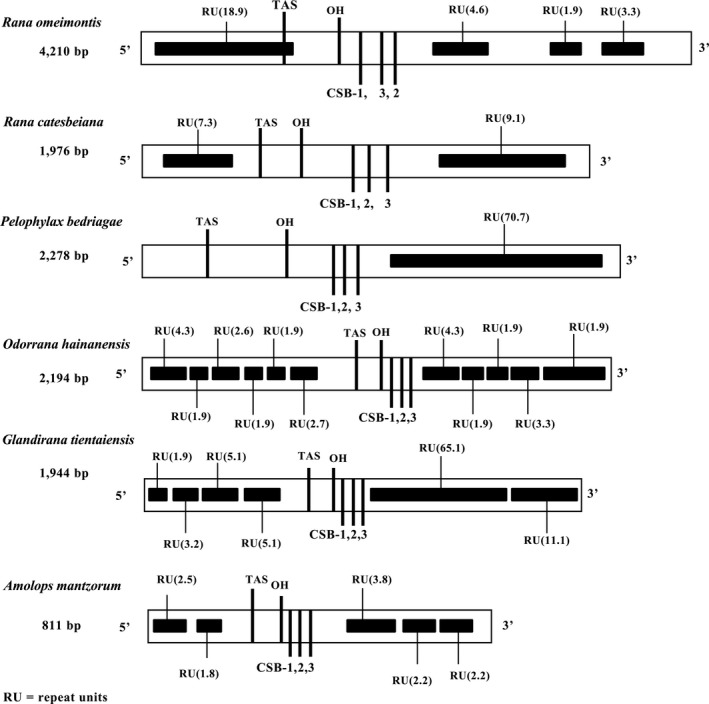
Main features of the mitochondrial control region in six Ranidae species. The location of features is shown in Table 4

A control region (CR) was found between the Cytb and ND5 gene (Table 2), which were researched detaily in *R. omeimontis* and analyzed by comprising with other ranids that were reported. CR region included typical structures: the termination‐associated sequence (TAS), H‐strand origin of replication (OH), and conserved sequence blocks (CSB‐1, CSB‐2, and CSB‐3) (Figure 5 and Table 4). CR region of *R. omeimontis* was 4,210 bp, which is extremely long among the vertebrates (Table 2). This can be explained by the fact that there were four distinct tandem repeat units located in the 5' and 3'‐sides of the CR region (Figure 5). 18 repeat units (RU) of 38 bp and one incomplete repeat unit (IRU) of 36 bp were found in the 5'‐end, while 4.6 RUs of 367 bp, 1.9 RUs of 30 bp, and 3.3 RUs of 15 bp in 3'‐end (Table 4). By comparing and analyzing the CR region of Ranidae species, location and order of several component characteristic of these species were more conservative (Figure 5 and Table 4). In addition, these conservative motifs in the CR were correlated with the transcription or replication of the mitogenome (Taanman, 1999). The repetitive units and number of repetitions varied greatly among different species of Ranidae (Figure 5 and Table 4), which are consistent with the larger variation of length in the region.

**TABLE 4:** Nucleotide sequences of conserved segments and tandem repeat units in the D‐loop region of six Ranidae species

Species	Segment	Start position	Stop position	Length (bp)	Sequence
*Rana omeimontis*	18.9 tandem repeat units	18	743	18 × 38+36	TCTATATGGATACTATCTCTATGTTTAATAATCATTAA
	TAS	701	717	17	ATTAATCTATATAGGTA
	OH	1,478	1542	65	AGGAATTTTAGGGGGATGCGATCCTCACCAACTTTAAGAAACCGTTCCTACTGCATATTAGTCAT
	CSB−1	1686	1,710	25	TCTTAATTAATGCTCGATTGACATA
	CSB−3	1807	1828	22	GTAATTGCCTTAAAACCCCCCC
	CSB−2	2,100	2,116	17	CCCCCCCTTTCCCCCCC
	4.6 tandem repeat units	2,116	3,808	4 × 367+225	CCATATCATATTGTAGTATATGTCATGTCACACCATATTGTACCACATCATATTGTGCCATATTGTATCATGCCAGATCATTTCGTGTAATACCCCATCATATCATGTTATATCCCGCCACACCGTCTCGTATATTGCTACATCATACCCAGTCATATCAAATTGCATCCCACTTTCCCGTTCTAGATCGTATCGAATTTTATCGCGTCGTGTCATTATATACAGCACACACCCAATTAAAGTAGTTCTGCCCCCGACATGTCATGCTCACCACACTATAATTTTATTACCAGATGCCACATATCGCACATGTTAAATCATACTGCATTGTGTCATGTCACATAGCCTCGTACCATACCGTGTTGTA
	1.9 tandem repeat units	3,849	3,905	1 × 30+27	CATGCGCACCTCCCTCATGCGCGCCTCCCT
	3.3tandem repeat units	3,857	3,905	3 × 15+4	CCTCCCTCATGCGCG
*Rana catesbeiana*	7.3 tandem repeat units	589	882	7 × 40+14	TAATATTATACTATTACTTATGTATAATCATCATCTATTA
	TAS	968	984	17	ATTAACAGTTATGTACA
	OH	1,860	1921	62	GGTAGTTTTTTTTGGGGTCCTTTCATCAGCTACTCCCAGTGGGCTCACTCCTAAACAACCGG
	CSB−1	2,107	2,131	25	TTTTAATTAATGTTAGATTGACATA
	CSB−2	2,232	2,248	17	CCCCCCCTTTCCCCCCC
	CSB−3	2,265	2,286	22	TAGTTTGCCTTAAAACCCCCCC
	9.1 tandem repeat units	2,466	2,565	9 × 11+1	TTTGTTTATAT
*Pelophylax bedriagae*	TAS	306	322	17	AATCCCCACTATATGTA
	OH	939	1,006	68	GGTATTTTTTTTTGGGGGGCCTTTCATCAGCTACTCACAGTGGGGACACGGCTTACGGTCAAGGTTAG
	CSB−1	1,057	1,081	25	TTTTAGTGAATGCTAGAATGACATA
	CSB−2	1,289	1,305	17	CCCCCCCTTTCCCCCCC
	CSB−3	1,324	1,345	22	TAGATTGCCTTGAAACCCCCCC
	70.7 tandem repeat units	1,454	2,584	70 × 16+11	CTATGAGTATCTATAC
*Odorrana hainanensis*	4.3 tandem repeat units	54	140	4 × 20+7	ATCATACATATATATACTTC
	1.9 tandem repeat units	55	167	1 × 60+53	TCATACATATATGTACTTCATCATACTATGTATAATCACCATTAATATATATAGTTACAT
	2.6 tandem repeat units	64	501	2 × 167+104	TATGTACTTCATCATACTATGTATAATCACCATTAATATATATAGTTACATTCATACATATATGTACTTCATCATACTATGTATAATCAGCATTAATATATATAATTGATCTCAAGATAAGCATTCTACTTATAACCACATAATATGTAAAATCTACATTATCACGG
	1.9 tandem repeat units	114	146	1 × 17+16	TTCATACATATATGTAC
	1.9 tandem repeat units	281	313	1 × 17+16	TTCATACATATATGTAC
	2.7 tandem repeat units	398	558	2 × 60+41	TCATACATATATGTACTTCATCATACTATGTATAATCACCATTAATATATATAGTTACAT
	TAS	422	439	18	AATCACCATTAATATATA
	OH	1,386	1,449	64	AATTCATCCCCACAGGGCCAGATCACGGGCATTAGTCCAAGGGTGGACATATTATGCAGCTGCA
	CSB−1	1,480	1504	25	TTTAAATGAATGCTCGAATGACATA
	CSB−2	1565	1581	17	CCCCCCCCTTCACCCAA
	CSB−3	1966	1988	23	TCTATCGCCCCAAGTATCGCCCC
	4.3 tandem repeat units	437	519	4 × 18+11	ATATAGTTACATTCATAC
	1.9 tandem repeat units	448	480	1 × 17+16	TTCATACATATATGTAC
	1.9 tandem repeat units	508	540	1 × 17+16	TTCATACATATATGTAC
	3.3 tandem repeat units	1968	2007	3 × 12+4	TATCGCCCCAAG
	10.6 tandem repeat units	2,122	2,248	10 × 12+7	TTTCTGCCTACG
Glandiranatientaiensis	1.9 tandem repeat units	33	366	1 × 180+154	CATATTAAGATGTACATATTATTCAAGACACATATTTATTAATGTATATAGATATATCTATGTATAATAACCATTCATCTAATTTATATACATATTAAGATGTACATATTATACAAGACACATATTTATTAATGTATATAGACATACCTATGTATAATAACCATTCATCTAATTTATATA
	3.2 tandem repeat units	33	321	3 × 90+19	CATATTAAGATGTACATATTATTCAAGACACATATTTATTAATGTATATAGATATATCTATGTATAATAACCATTCATCTAATTTATATA
	5.1 tandem repeat units	66	299	5 × 48–6	ATTTATTAATGTATATAGATATATCTATGTATAATAACCATTCATCTA
	5.1 tandem repeat units	92	316	5 × 40+25	ATGTATAATAACCATTCATCTAATTTATATACATATTAAG
	TAS	660	676	17	CTTAACAATTTTATGTA
	OH	1,199	1,260	62	ATTTTTCTTTTGGGGGGAGATCTCAACCAGCATCTCCAGTGGGCCCACGACATATAGTCCAC
	CSB−1	1,317	1,341	25	TAAAAATGAATGCTAGATTGACATA
	CSB−2	1,444	1,460	17	CCCCCCCTTTCCCCCCC
	CSB−3	1,478	1,500	23	TAGATTTGCCTTAAAACCCCCCC
	65.1 tandem repeat units	1576	2,196	65 × 10–29	TATACCCATA
	11.1 tandem repeat units	1795	1981	11 × 16+11	ATATACACATATATAC
*Amolops mantzorum*	2.5 tandem repeat units	176	394	2 × 89+41	TTATAATGTAATGCCCAATACCTATATATGTATAATAACCATAAATTTATATGCACCATATTCAAAATCACCATATTATGCTTCATAAA
	1.8 tandem repeat units	239	691	1 × 248+205	AAAATCACCATATTATGCTTATAAATTATAATGTAATGCCCAATACCTATATATGTATAATAACCATAAATTTATATGCACCATATTCAAAATCACCATATTATGCTTCATAAATTATAATGTAATGCACAACAACTATATATATATAATAACCAAATTCAAAATCACCATATATTAAATTAACCATAATGTATGCTTCATAACTATCAATATATATAATAACCATAAAACTAAAATAATCATAATCT
	TAS	419	435	17	ATTAACCATAATGTATG
	CSB−1	713	737	25	TATTAATATATAATAATCATAAATT
	OH	1726	1797	72	GGTATTTTTTTTTTGGGGAGCTTTCACCTGGCAACTCAAGTGGGTTCACGACATATAGTCCGGGTTGGACAT
	CSB−2	1938	1954	17	CCCCCCCTTTCCCCCCC
	CSB−3	2058	2079	22	CAAACTTCCATAAAAACCCCCC
	3.8 tandem repeat units	452	788	3 × 87+76	ATATATAATAACCATAAATTTATATATACCATATTTAAAATTACCATATTATGCTTCATAATTATAATGTAATGCATATAACTATTA
	2.2 tandem repeat units	648	680	2 × 15+3	ATTTAAATATACCAT
	2.6 tandem repeat units	710	987	2 × 109+60	AACTATTAATATATAATAATCATAAATTTATATACAACACATTAAGATTAACATATTAAAGCTACATATACTATAATGTATGTATAAAGAAATTATATGTATGCTTAAA

### 3.5 Mitogenome organization in ranid frogs

**FIGURE 6:**
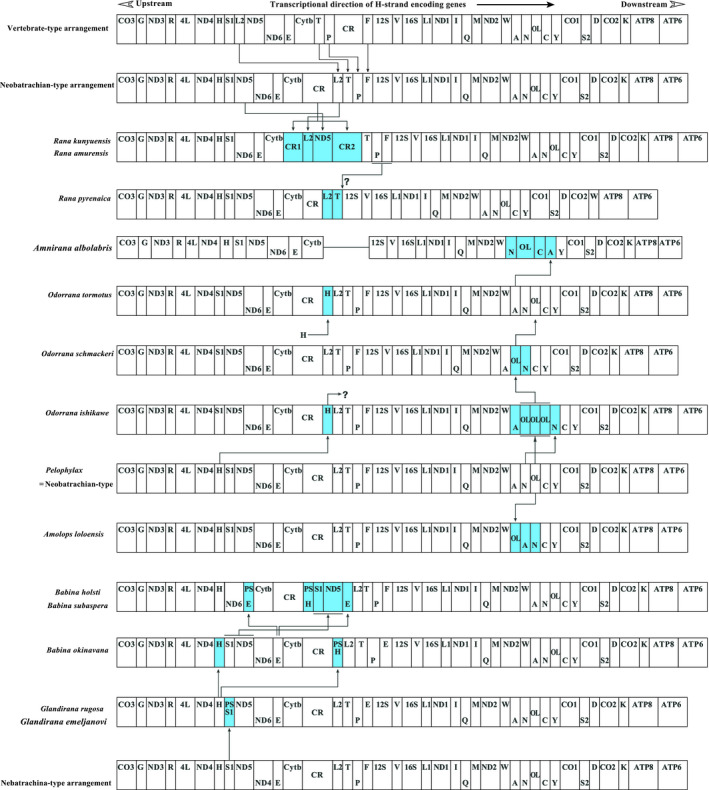
Comparison of mt gene arrangements among ranid taxa. The transcriptional direction of H‐strand encoding genes and the upstream and downstream notations used in this article are shown by a closed arrow and closed arrowheads, respectively. The H‐ and L‐strand encoded genes are denoted at the top and bottom of each gene box, respectively. The sizes of the boxes do not reflect actual gene length. Closed arrows show the rearranged genes and infer the evolutionary directions of the rearrangements (see the text). Transfer RNA genes (trns) are designated by single‐letter amino acid codes. L1, L2, S1, and S2 indicate trns for Leu (UUR), Leu (CUN), Ser (AGY), and Ser (UCN), respectively. Trn boxes with “ps” indicate the pseudogenes. The control region is abbreviated as CR. OL indicates the region of the light‐strand replication origin including a typical stem‐loop structure. Other genes are abbreviated as follows: 12S and 16S, 12S, and 16S ribosomal RNA; CO1‐3, cytochrome c oxidase subunits 1–3; Cytb, cytochrome b; ND1‐6 and 4L, NADH dehydrogenase subunits 1–6 and 4L

According to our comparison of mtDNA genome organization, the gene rearrangement among the 48 Ranidae mitogenomes revealed ten gene rearrangement types (type I ‐ type X, Figures 6 and 8). In addition to the genus *Pelophylax* and *Hylarana* (the neobatrachian‐type; Figure 6), these rearrangements are scattered in different genera (*Rana*, *Amolops*, *Odorrana*, *Babina*, *Glandirana,* and *Amnirana*) of the family Ranade. Similar to most of species in neobatrachians, *R. omeimontis* show common modified gene arrangement (the neobatrachian‐type; Figure 6). Additionally, this rearrangement pattern was the most basic type in the neobatrachians, and on this basis, another ten novel rearrangement types (from Type I to Type XI) are generated by different rearrangement ways (Figure 6). In genus Rana, the trnP and trnF gene loss was only discovered in Type IX (*R. pyrenaica*), while *R. kunyuensis* and *R. amurensis* had a consistent arrangement Type X. This type revealed more complex structure than that of Type IX: the ND5 gene was translocated from the trnS2 (AGY) downstream to the trnL2 (CUN) downstream, and one additional CR region was inserted into the upstream of TPF gene cluster and finally forming a particular trnS2‐ND6‐trnE‐Cytb‐CR‐trnL2‐ND5‐CR‐trnF order (Figure 6). In conclusion, there are various rearrangement types in Ranidae.

### 3.6 Phylogenetic relationships

In our study, the concatenated PCG data of the mitogenome sequences contained 11,235 nucleotide positions (4,485 conserved sites, 6,750 variable sites, and 6,137 potentially parsimony‐informative sites). BI and ML methods consistently support similar tree topologies with strong node‐supporting values (BPP = 1.00; bootstrap values (BS) ≥ 58%) (Figure 8); while using another dataset, except for two species (*Babina holsti* and *B. subaspera*), the phylogenetic tree constructed is consistent with the topology obtained by using 13 protein‐coding genes. In the BI tree, *B. holsti* and *B. subaspera* gather in the same branch and form sister groups with three other species ((*B. holsti* + *B. subaspera*) + (*B. okinavana* + (*B. adenopleura* + *Hylarana guentheri*))), while its topological structure was recovered as (*B. holsti* + (*B. subaspera* + (*B. okinavana* + (*B. adenopleura* + *Hylarana guentheri*)))) based on ML tree, and *B. holsti* was the basal position of the clades (Figures S2 and S3).

The topology obtained based on the two datasets is very similar, and they both support the following classification and phylogenetic relationships of Ranidae (Figure 8, Figures S2 and S3): A) the clade group (*Babina* + (*Rana* + *Odorrana*)); B) the monophyly of *Rana*, *Odorrana*, *Amolops*, *Pelophylax,* and *Glandirana*; C) the clade group of *Pelophylax* + *Amolops*; D) the paraphyly of genus *Babina* and *Hylarana guentheri* is embedded in it; E) the subbasal lineage of the genus *Glandirana*; and F) *Amnirana albolabrics* as the most basal lineage of species classification of Ranidae. In *Rana*, the data strongly support the monophyly of *Rana* (PPS 1.00 in BI, BS 100% in ML) (Figure 8). *R. omeimontis*and *R. chaochiaoensis*are clustered on the same branch, which indicates a closer relationship.

## 4 DISCUSSION

### 4.1 Characteristics analysis of the mitogenomes

The *R. omeimontis* mitogenome possessed the same genomic arrangement with those of *R. chaochiaoensis*, *R. draytonii*, *R. dybowskii*, *R. kukunoris,* and *R. huanrensis* (Dong et al., 2016; Li, et al., 2016; Li, et al., 2016; Wang et al., 2020), and this genomic arrangement belongs to the typical neobatrachian‐type (Dong et al., 2016; Kurabayashi et al., 2010). The length and base composition of complete mitogenomes among *Rana* species mainly depended on the number of noncoding control region (D‐loop) and tandem repeat unit in this region (Dong et al., 2016; Li, et al., 2016; Li, et al., 2016; Wang et al., 2020). Also, to the D‐loop region, the mitochondrial genome of *R. omeimontis* is similar to that of the published species in gene arrangement and base composition (Yang et al., 2018). In this study, D‐loop region possessed four distinct tandem repeat units and a long sequence (367 bp) of tandem repeat units (Table 4), and however, the sequence submitted by Yang et al. (2018) had only one tandem repeat unit type (35 repeat units of 38 bp), and resulting in a large change in the length of the region. Therefore, the analysis of *R. omeimontis* shows that there is a big genetic variation and length change in this region.

### 4.2 Extensive gene rearrangement in Ranidae

The gene arrangement of the metazoan mitogenome is usually conserved (Boore, 1999) and the gene rearrangements are comparatively rare or random (Yang et al., 2018). Most archaeobatrachian show the vertebrate‐type gene arrangement pattern. However, the neobatrachian species appear in various types of mitogenome reorganization especially for ranids species (Kurabayashi et al., 2010; Yang et al., 2018). Here, we summarized ten gene arrangement patterns of Ranidae species (Figures 6 and 8), and these rearrangements mainly occurred at the two regions (ND4‐trnF and trnW‐COI region). In genus *Rana*, the gene arrangement of *R. omeimontis* mtDNA is in accord with the neobatrachian‐type. This is a case for *Caudata* species (Xia et al., 2010). Yet in *R. kunyuensis* and *R. amurensis*, the ND5 is translocated from ND6 upstream to trnL2 downstream, and the copied D‐loop was transferred into the upstream of the trnT (Kumazawa et al., 1998; Kurabayashi et al., 2008), which form a trnLeu‐ND5‐D‐loop region together. In *Gymnophiona* mitochondrial genomes, the gene rearrangements were observed at the trnW‐CO1 region (San Mauro et al., 2006). Moreover, for genus *Odorrana* species, only *O. ishikawae* species show gene arrangement pattern of WAO_L_NCY, and the O_L_ region repeats three times. The derived trnH position has also been detected (Figure 6). Conversely, the other species do not appear in this gene arrangement pattern (Kurabayashi et al., 2010). Moreover, in genus *Amolops*, the *A. mantzorum* group is identical with the neobatrachian‐type, while the *A. ricketti* group the positions of the O_L_ is translocated from its typical WANO_L_CY to WO_L_ANCY trn cluster. Such arrangement is consistent with their phylogeny (Figure 8). For this phenomenon, we speculate that the O_L_ rearrangement pattern was the recognition characteristics of th*e A. mantzorum* group and the *A. ricketti* group (Figure 8). For Anura, rearrangements of mitogenome mainly occurred at three sites including the D‐loop region, OL region, and the IQM genes (Li et al., 2010). Moreover, for OL region, in some Ranidae mitochondrial genomes, we found a lot of rearrangement pattern, namely WAO_L_O_L_O_L_NCY, WO_L_ANCY, WNO_L_CAY, and WAO_L_NCY (Figure 6). From the above analysis, we speculated the ND4‐trnF and trnW‐CO1 region should be the frequent occurrence area of mitogenome rearrangement in Ranidae (Yang et al., 2018).

About the genus *Glandirana*, the trnS2 (AGY) of *G. emeljanovi* and *G. rugosa* is translocated from the typical location of trnH downstream, and a trnS2 pseudo has been formed in its original region. The real trnS2 of the two species has not been discovered from the examined region (Kurabayashi et al., 2010). But in genus *Babina*, the trnH‐trnS2‐ND5 region of *B. holsti* and *B. subaspera* is translocated from the original ND6 upstream region to the derived CR downstream, but the rearranged trnH develops into a pseudogene and real trnH maintains at the original region. The trnE is moved from the typical Cytb upstream position to the LTPF gene cluster upstream and the original position of the trnE has become a pseudo gene. This phenomenon occurs in many amphibians (Atsushi & Masayuki, 2013; Irisarri et al., 2012; Sumida et al., 2001; Xia et al., 2014). Based on comparing the gene arrangements among the 48 complete mitogenomes known, ten types of gene arrangement were analyzed and presented in Figures 6 and 8. Therefore, unexpected diversity of mtDNA gene organizations occurs in ranid frogs.

Interestingly, *R. amurensis* and *R. kunyuensis* possessed two duplicate D‐loop regions and this phenomenon was also found in other frogs (Afrobatrachia frogs, Kurabayashi & Sumida, 2013; *A. larutensis*, Kurabayashi et al., 2010; *Hoplobatrachus* spp., Yu et al., 2012; Yu et al., 2012; Mantellidae frogs, Kurabayashi et al., 2008; *Rhacophorus schlegelii*, Sano et al., 2005). Wang et al. (2015) revealed that the duplicated D‐loop regions were similar to original D‐loop structure, which will result from homologous recombination between paralogous D‐loop regions. However, in this study, the formation mechanism of duplicated D‐loop regions of this genus *Rana* still needs further study and discussion.

**FIGURE 7:**
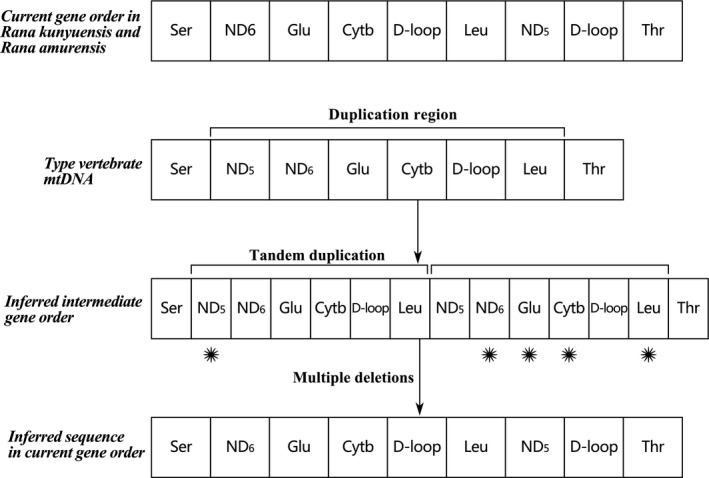
A model for gene reorganization in the mtDNA genomes of *R. omeimontis*, *R. kunyuensis* and *R. amurensis*. After tandem duplication of a gene region is produced, multiple deletions of redundant genes occur. The gene order of *R. omeimontis* is same as neobatrachians’ general one. Asterisks denote random missing

According to the traditional reasoning that animal mitogenomes lack DNA recombination events, mtDNA gene rearrangement has been considered to result from the tandem duplication and then the deletions of redundant genes (tandem duplication and random loss, TDRL pattern) (San Mauro et al., 2006; Shi et al., 2015; Tom et al., 2018; Xia et al., 2016). In addition, researchers agree well with the recombination model between upstream regions of duplicated the control regions (Kurabayashi et al., 2008; Sammler et al., 2013; Zhou et al., 2016). Subsequently, gene transfers via retrotransposition may be a pattern of gene rearrangement in animal mtDNAs (Endo et al., 2005; Han & Hahn, 2012). In genus *Rana*, gene rearrangement of the *R. kunyuensis* and *R. amurensis*can belong to the TDRL model. The two species may have undergone a replication and a random deletion event, which resulted in the current rearrangement (Figure 7). So more experiments and reasoning are needed to confirm the gene rearrangement in the family Ranidae and genus *Rana*.

### 4.3 Molecular phylogenetic analysis in Ranidae

**FIGURE 8:**
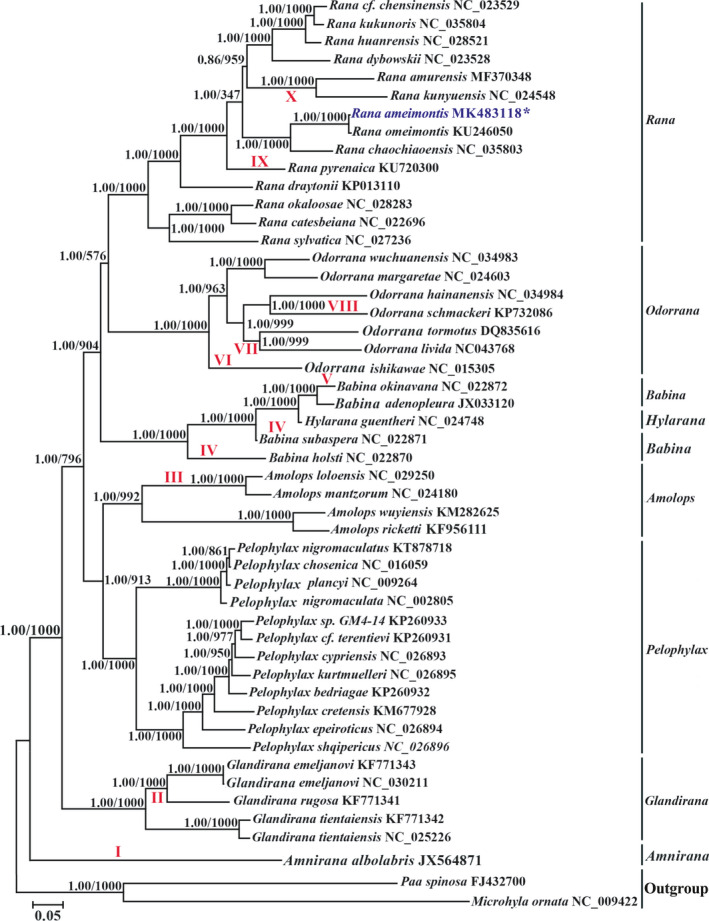
Phylogenetic tree of the relationships among 48 mitogenome sequences of Ranidae and two species of Microhylidae and Dicroglossidae as outgroup (*Microhyla ornata* and *Paa spinosa*) based on the nucleotide dataset of the 13 mitochondrial protein‐coding genes of 11,235 nucleotides. Branch lengths and topology are from the BI analysis. Numbers above branches specify posterior probabilities from Bayesian inference (BI) and bootstrap percentages from maximum likelihood (ML, 1,000 replications) analyses. The GenBank numbers and scientific names of all species are shown in Table 1. Tree topologies produced by Bayesian inferences (BI) and maximum likelihood (ML) analyses were equivalent. Bayesian posterior probability and bootstrap support values for ML analyses are shown orderly on the nodes. The mt genomic rearrangement characteristics of each species on the tree are as follows: I. The positions between trnA and trnN‐OL‐trnC were exchanged accompanied by the insertions of a noncoding regions and finally forming the new trnW‐trnN‐OL‐trnC‐trnA‐trnY order; II. trnS2 (AGY) pseudogene; III. translocation of OL (between trnW and trnA); IV. CR‐psH‐S1‐ND5, translocation of trnH, trnS1, and ND5 (Insert the back of the CR, and trnH of the original location becomes a pseudogene); translocation of trnE (LTPF before); V. trnH pseudogene between CR and L2; VI. exchanging trnN and OL positions, triplication of OLs, translocation of trnH (trnH between CR and LTPF cluster); VII. translocation of trnH (LTPF before); VIII. exchanging trnN and OL positions, trnH gene loss; IX. TrnP and trnF gene loss; and X. translocation of ND5 and a copy of the CR are inserted together between trnL2 and trnT (D‐loop‐trnL2‐ND5‐D‐loop‐trnT).The asterisks indicate new sequences generated in this study

We analyzed the structures of 50 mitogenome sequences and explored the phylogenetic relationships among the species of genus *Rana* and other ranids using 13 PCGs and 13 PCGs + 2 rRNAs + 21 tRNAs nucleotide datasets. Totally, in this study the genus level phylogeny placement of Ranidae species was consistent with the topological trees from Bu et al. (2016), Li et al. (2014) and Yang et al. (2018), but inconsistent with the results of other researchers (Wiens et al., 2009; Pyron& Wiens, 2011). Our phylogenetic analyses revealed that the monophyly of the five genera (*Amolops, Glandirana*, *Rana, Odorrana* and *Pelophylax*) was well supported (Matsui et al., 2006; Xue et al., 2016) (Figure 8, Figure S2), but the paraphyly of *Babina* is supported (Ni et al., 2016), which is different with Kakehashi et al. (2013). Phylogenetically, genus *Amolops* becomes the sister taxon of *Pelophylax*. And the four species in genus *Amolops* are divided into *A. mantzorum* + *A. loloensis* and *A. ricketti* + *A. wuyiensis* clades, namely the *A. mantzorum* clade and the *A. ricketti* clade, respectively (Figure 7), supporting the previous study results (Matsui et al., 2006; Ngo et al., 2006). In addition, the two groups can be distinguished by gene rearrangement (Figure 6). Genus *Babina* form the sister taxon to the *Rana* + *Odorrana* group, which is similar to the results of Ni et al. (2016) and Xue et al. (2016). For the genus *Glandirana*, it is located on the subbasal position of several other genera (BPP = 1.00, BP = 100%), and this is also in accord with the results of Bu et al. (2016) and Yang et al. (2018), but it is different with other research results that supported the *Glandirana* in an embedded position within the Ranidae topological tree with weak support value (Che et al., 2007; Kurabayashi et al., 2010; Xia et al., 2010, 2014). By analyzing previous studies, the results show that genus *Rana* and *Babina* are regarded as the sister genera of *Odorrana*. Che et al. (2007), Wiens et al. (2009), Kurabayashi et al. (2010) and Xia et al. (2014) found genus *Babina* was the sister genera of *Odorrana* with the single gene or very few genes, and subsequently, Kakehashi et al. (2013) obtained the same system taxonomic status with two rRNAs and 13 PCGs.

Our phylogenetic results strongly supported that genus *Rana* as the sister taxon of *Odorrana* and were in accordance with other research results (Bu et al., 2016; Xue et al., 2016). Furthermore, Kakehashi et al. (2013) also put forward that the species of genus *Babina* gathered in a monophyletic clade. But, the *H. guentheri*, as reported by Ni et al. (2016) and Yang et al. (2018), was embedded in genus *Babina* group in our topological trees. The phylogenetic relationship of *H. guentheri* was comparatively complicated (Wu et al., 2016). Previous studies have shown that this species was once put into several different genera, namely *Rana*, *Hylarana*, *Sylvirana*, *Hylorana,* and *Boulengerana* (Frost, 2017; Oliver et al., 2015). Our phylogenetic results supported that *H. guentheri* were nested within genus *Babina* clade, which in turn revealed the paraphyly of the genus *Babina*. Analysis indicates that *H. guentheri* may actually belong to the genus *Babina* (Figure 8). However, it is needed to clarify the evolutionary position of this species in the future. The phylogenetic relationship of the whole family Ranidae is (*Glandirana* + ((*Pelophylax* + *Amolops*) + ((*Rana* + *Odorrana*)*+ Babina*))), supporting the results of Kakehashi et al. (2013), Xue et al. (2016), and Yang et al. (2018), but different from the results of Ngo et al. (2006) and Ni et al. (((*Pelophylax* + *Amolops*) + (*Glandirana* + ((*Rana* + *Odorrana*) + *Babina*))) (Figure 8, Figure S2 and S3) (2016).

## 5 CONCLUSIONS

We analyzed and determined the mitogenome sequence of *R. omeimontis*, and found it is similar to other vertebrates with many significant features including a codon usage bias, non‐Watson‐Crick base pairs among the tRNA secondary structures, and VNTR (Variable number tandem repeat) in the control region and, etc. Some mitogenome arrangements can reflect phylogenetic relationships (Irisarri et al., 2012; Kurabayashi et al., 2008). In current research, the phylogenetic status was analyzed, by rebuilding topological trees (ML and BI) with 13 PCGs and 13 PCGs + 21 tRNAs + 2 rRNAs nucleotide datasets, we found that *R. omeimontis* was closely related to *R. chaochiaoensis* compared to the *R. amurensis* and *R. kunyuensis*, and we also explored there was a new way of gene rearrangement (ND6‐trnE‐Cytb‐D‐loop‐trnL2‐ND5‐D‐loop). The rearrangement pattern can be used as the recognizing marker of *R. amurensis* and *R. kunyuensis*. The formation mechanism of this rearrangement type needs further study in the future.

## CONFLICT OF INTEREST

The authors declare that there are no conflicts of interest.

## AUTHOR CONTRIBUTION

**Lichun Jiang:** Project administration (equal); Validation (equal); Writing‐original draft (equal). **Min Zhang:** Data curation (equal); Formal analysis (equal); Investigation (equal). **Lu Deng:** Data curation (equal); Formal analysis (equal). **Zhongwen Xu:** Data curation (equal); Investigation (equal); Software (equal); Visualization (equal). **Hongyan Shi:** Data curation (equal); Formal analysis (equal); Methodology (equal). **Xiaodong Jia:** Data curation (equal); Formal analysis (equal); Software (equal). **Zhenli Lai:** Data curation (equal); Investigation (equal); Methodology (equal); Software (equal); Visualization (equal). **Qiping Ruan:** Funding acquisition (supporting); Project administration (lead). **Wei Chen:** Funding acquisition (equal); Investigation (lead); Writing‐review & editing (equal).

## Supporting information

Fig S1

Fig S2

Fig S3

Table S1‐S2

## Data Availability

The following information was supplied regarding the deposition of DNA sequences: GenBank accession numbers: MK483118.
