# Supplementary figures and images for: Characteristics of the mitochondrial genome of Rana omeimontis and related species in Ranidae: Gene rearrangements and phylogenetic relationships
Source: Ecol Evol. 2020 Oct 31;10(23):12817–37. doi: 10.1002/ece3.6824 (PMC7713938; doi:10.1002/ece3.6824)

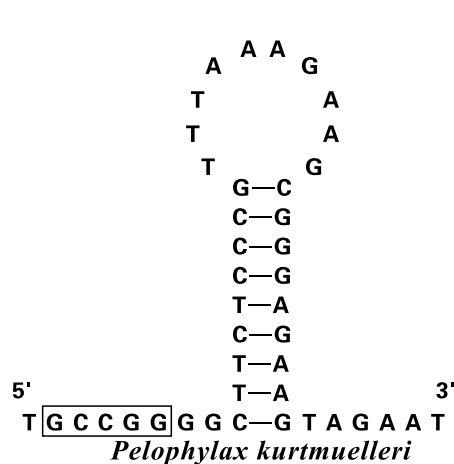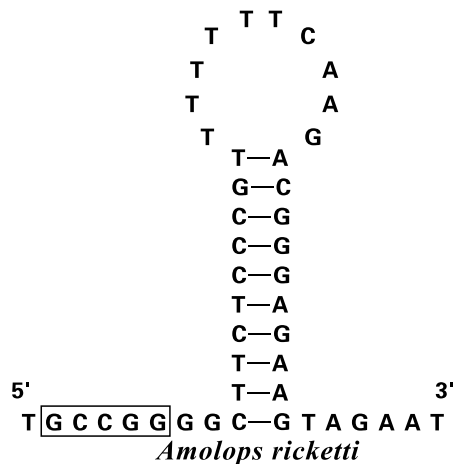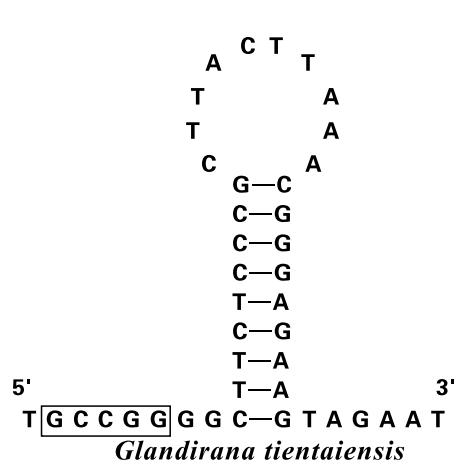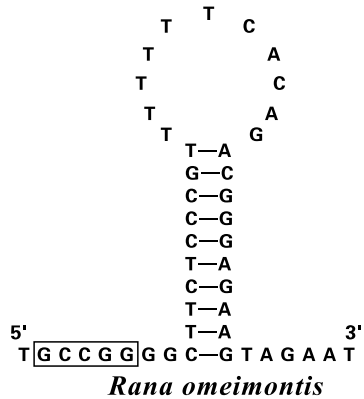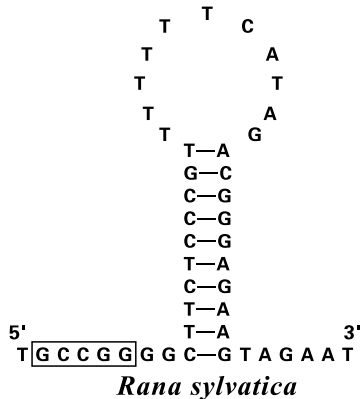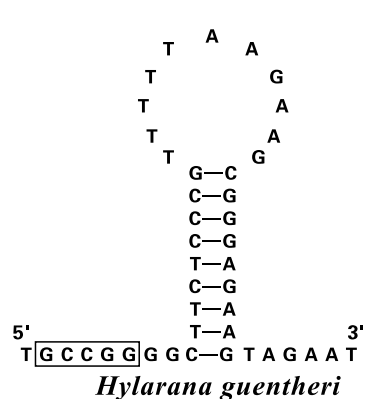

Supplement: Supplementary file 1 — Fig S1 [file ECE3-10-12817-s001.pdf]

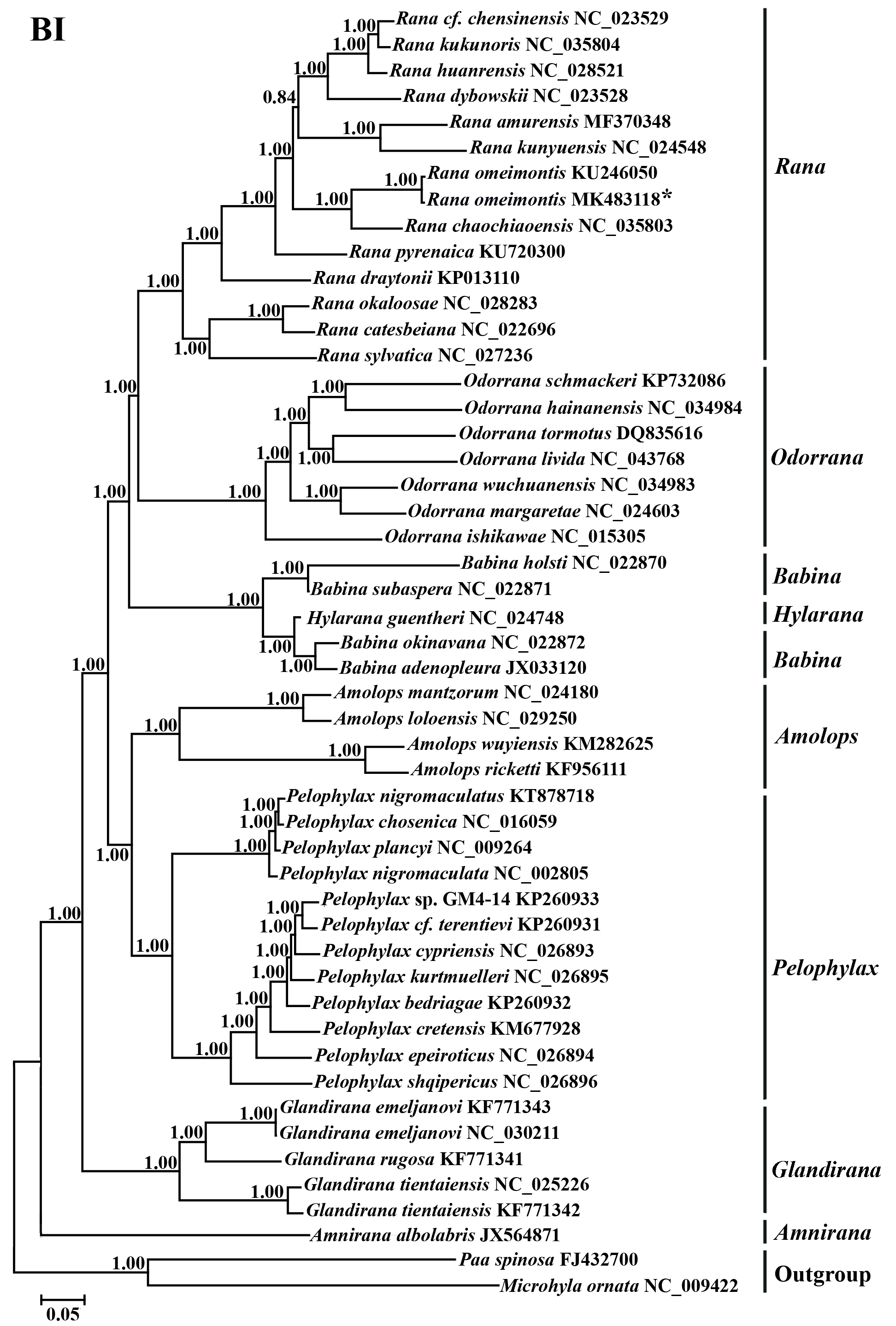

Supplement: Supplementary file 2 — Fig S2 [file ECE3-10-12817-s002.tif]

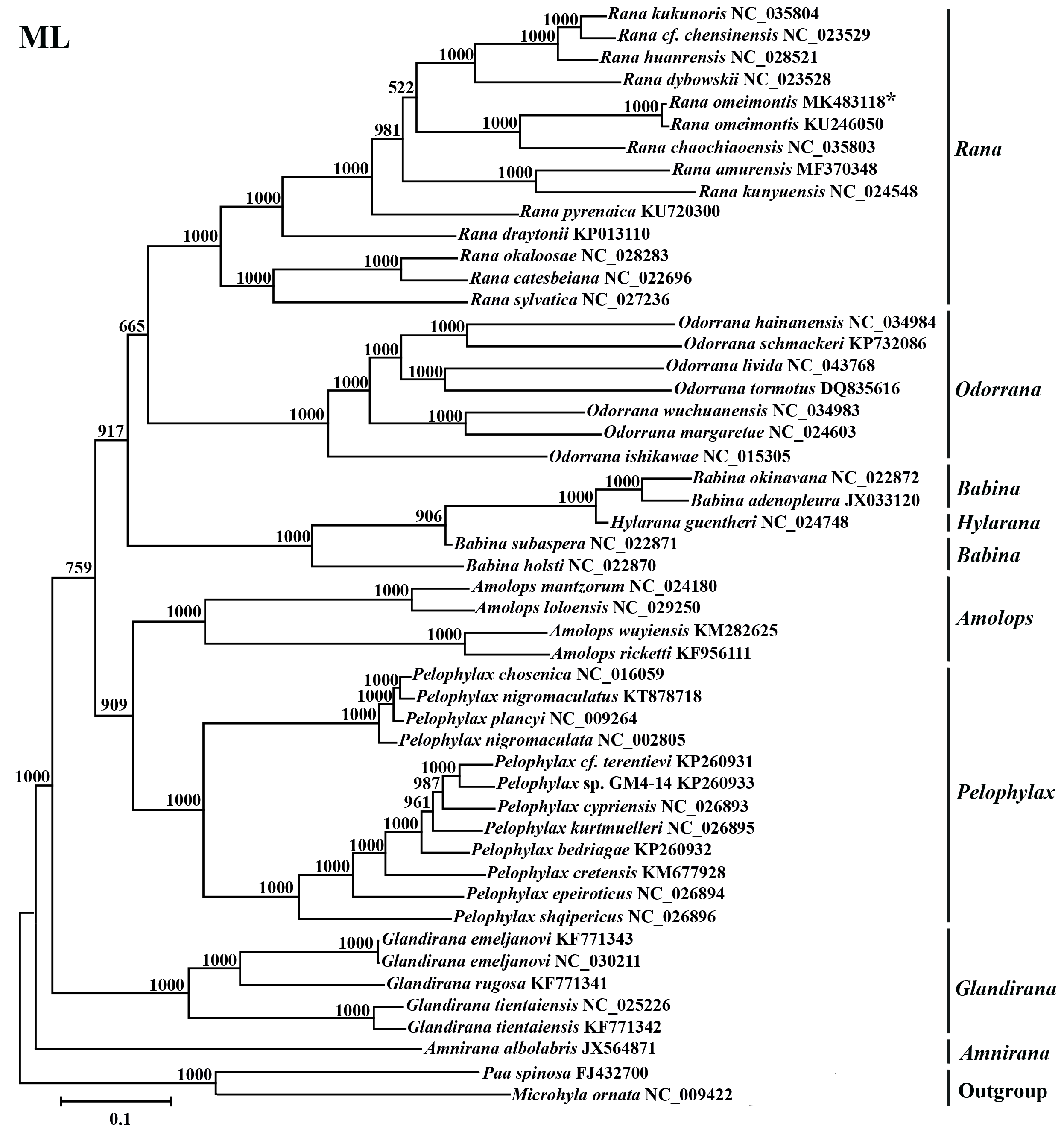

Supplement: Supplementary file 3 — Fig S3 [file ECE3-10-12817-s003.tif]
