# Supplementary material for: Characteristics of the mitochondrial genome of Rana omeimontis and related species in Ranidae: Gene rearrangements and phylogenetic relationships
Source: Ecol Evol. 2020 Oct 31;10(23):12817–37. doi: 10.1002/ece3.6824 (PMC7713938; doi:10.1002/ece3.6824)
Supplement: Supplementary file 4 — Table S1‐S2 [file ECE3-10-12817-s004.docx]

**TABLE S1** Base composition for the 13 PCGs in the *Rana omeimontis* mitogenomes.

| Species | A(%) | T(%) | C(%) | G(%) | A+T(%) | C+G(%) | AT-skew | GC-skew |
| --- | --- | --- | --- | --- | --- | --- | --- | --- |
| *Rana omeimontis* | 24.35 | 29.63 | 30.08 | 15.94 | 53.98 | 46.02 | -0.10 | -0.31 |
| 1st | 26.71 | 23.20 | 25.49 | 24.61 | 59.91 | 40.10 | 0.07 | -0.02 |
| 2nd | 18.00 | 41.23 | 27.64 | 13.13 | 59.23 | 40.77 | -0.51 | -0.36 |
| 3th | 28.34 | 24.47 | 37.10 | 10.09 | 52.81 | 47.19 | 0.08 | -0.57 |

**TABLE S2** (A+T) content of 13 PCGs in the *Rana amurensis* mitogenomes.

| Gene | ATP6 | ATP8 | COX1 | COX2 | COX3 | Cytb | ND1 | ND2 | ND3 | ND4 | | ND4L | ND5 | ND6 |
| --- | --- | --- | --- | --- | --- | --- | --- | --- | --- | --- | --- | --- | --- | --- |
| *Rana amurensis* | **56.01** | 59.88 | 53.20 | 54.22 | 51.91 | **51.10** | 55.17 | 54.98 | 54.12 | | 55.07 | 52.63 | 54.87 | 51.92 |
